# Mapping the unmappable—Rapid high-density contact mapping in hemodynamically unstable ventricular tachycardia using a novel star-shaped multipolar catheter

**DOI:** 10.1016/j.hrcr.2023.07.021

**Published:** 2023-08-03

**Authors:** Johanna B. Tonko, Simon Sporton, Vinit Sawhney, Mehul Dhinoja

**Affiliations:** ∗ St Bartholomew’s Hospital, London, United Kingdom; † Institute for Cardiovascular Science, University College London, London, United Kingdom

**Keywords:** Ventricular tachycardia, Hemodynamic instability, Multipolar mapping catheter, Electroanatomical mapping system, Local activation time, Detection algorithms, Unipolar signal, Substrate mapping, Activation mapping

**Key Teaching Points**

• Hemodynamically unstable and/or alternating, irregular, or nonsustained ventricular tachycardias (VT) are notoriously difficult to map with sequential contact mapping systems, and ablation strategies are often limited to substrate-guided approaches.
• New multipolar catheter designs with numerous small electrodes covering a wide surface area and using a tightly referenced indifferent electrode in the shaft to minimize far-field interference open new possibilities for efficient, rapid identification of critical areas to target for ablation.
• In diseased myocardium, reliable automated detection and local activation time annotation of high-density, low-amplitude, fractionated near-field signals in presence of far-field signals remains an unresolved challenge for scar-related substrate and VT mapping even in advanced mapping systems.

## Introduction

Percutaneous catheter ablation for ventricular tachycardia (VT) in structural heart disease is an important adjunctive treatment option to reduce overall VT burden and recurrence, implantable cardioverter-defibrillator (ICD) shocks, and hospitalization by eliminating or excluding the arrhythmogenic substrate.^1^ Yet, despite encouraging results with a trend toward improved survival in registry data following VT ablation^2^ as well as improvements in preprocedural substrate characterization and high-density electroanatomical 3D mapping, procedure times remain comparatively long, complication rates remain significant, and success rates for certain VT subtypes are, at best, moderate. Crucial procedural aspects of how rapidly and accurately to identify all critical areas to target for ablation without prolonged VT induction and hemodynamic compromise remain an area of intense debate. Hemodynamically unstable, alternating, nonsustained, and irregular VTs in particular represent a major challenge for invasive activation mapping. Multiple strategies for substrate mapping have been suggested,^3^ but correct detection and annotation of multiple low-amplitude, fractionated signals close to the noise floor and overshadowed by larger far-field signals remains a challenge.

Innovative mapping catheter designs have been developed to overcome these limitations. The star-shaped 48-microelectrode Octaray^TM^ catheter has shown increased mapping density while reducing mapping time as compared to the Pentaray^TM^ when applied in the atria.^4^^,^^5^ Experience of its use in the ventricle is scarce and predominantly limited to animal studies.^6^

We present a scar-related VT case using the new Octaray mapping catheter (Biosense Webster, Inc., Irvine, CA), highlighting advantages and limitations of this new mapping tool.

## Case report

We report the case of a 71-year-old female patient with dilated cardiomyopathy and severe left ventricular dysfunction, broad left bundle branch block, and a secondary-prevention cardiac resynchronization therapy defibrillator in situ. She was known to have recurrent VTs, including an electrical storm managed conservatively 2 months prior to the current episode. She presented at her local hospital with 25 appropriate ICD shocks following acceleration of the VT by antitachycardia pacing into the VF zone, with 1 shock failing to terminate the VT, and 314 pace-terminated VT episodes with ventricular cycle lengths between 360 and 440 ms. A total of 4953 nonsustained VT episodes were also documented, reducing her effective biventricular pacing percentage to 77%. No acute reversible trigger was identified. The 12-lead electrocardiogram during VT showed an irregular broad complex tachycardia with QRS width of 120 ms and morphology suggesting an inferoseptal exit. The VT remained refractory to maximal medical therapy, including trials of procainamide, amiodarone, lidocaine, nadolol, and general anesthesia as well as overdrive pacing at 100 beats per minute. Given her clinical presentation and failed response to medical management, she was transferred while ventilated to our hospital for VT ablation.

### Invasive mapping and ablation procedure

**Figure 1:**
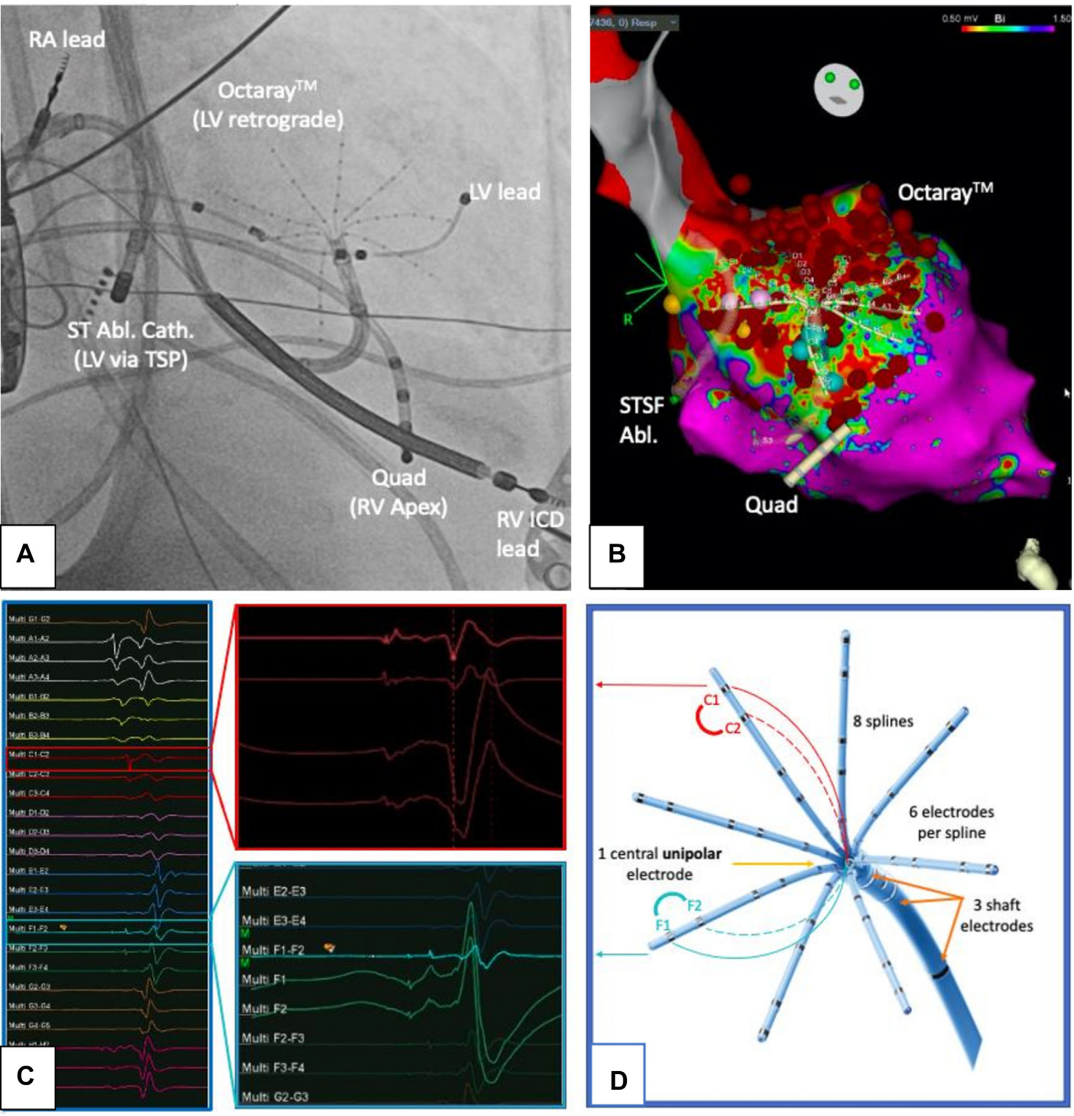
**A:** Intraprocedural setup in anteroposterior view: ST ablation catheter to left ventricle (LV) via trans-septal puncture (TSP) and Octaray (Biosense Webster) retrograde via aortic valve, quadripolar catheter in right ventricle (RV). Note cardiac resynchronization therapy defibrillator in situ and quadripolar electrophysiology catheter in RV apex. **B:** Corresponding 3D CARTO model of substrate map with large basal-midventricular scar on the septum in the bipolar map with multiple ablation lesions (*dark red balls and circles*), blue lesions representing tagged late potentials during substrate mapping, yellow lesions representing conduction system signals. **C:** Bipolar electrogram (EGM) during ventricular tachycardia of all 8 Octaray splines with magnification of selected bipolar EGMs with respective TRUEref unipolar EGMs using the central isolated shaft electrode as indifferent pole. Sharp, clear local potential on unipolar EGM with no relevant baseline drift. **D:** Octaray catheter, here shown with 2-5-2-5-2 electrode configuration.

**Figure 2:**
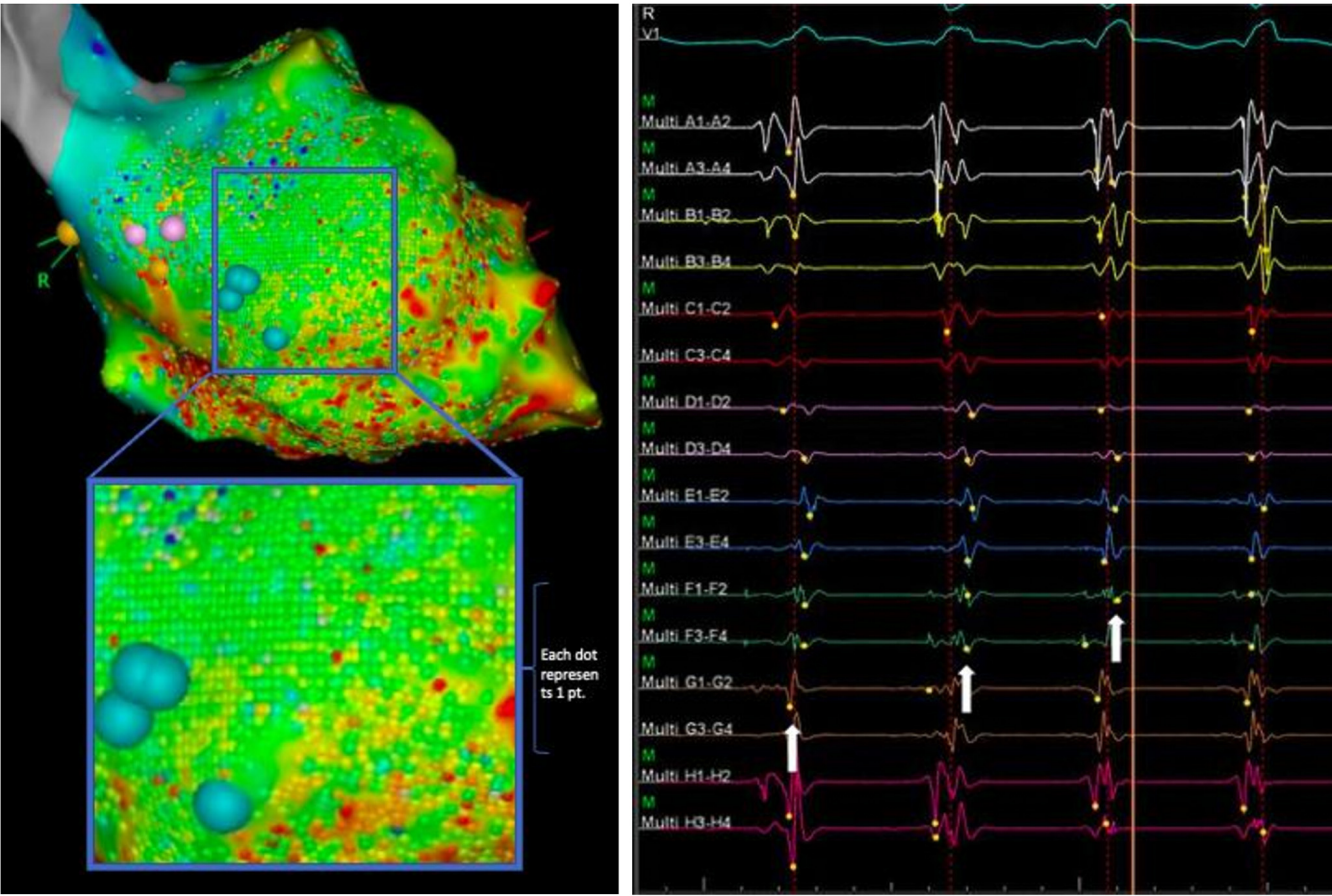
Left panel shows ultra-high-density mapping with acquisition of >17,400 points in only 24 minutes for left ventricular substrate and activation mapping in ventricular tachycardia (VT). Each dot represents an acquisition point projected on the shell at maximum density setting. Magnification shows that nearly the entire area of interest is mapped with the maximum point density allowed by the system. Right panel shows wavefront algorithm during VT with presystolic potentials, with intermittent failure to annotate the low-amplitude near-field potentials.

**Figure 3:**
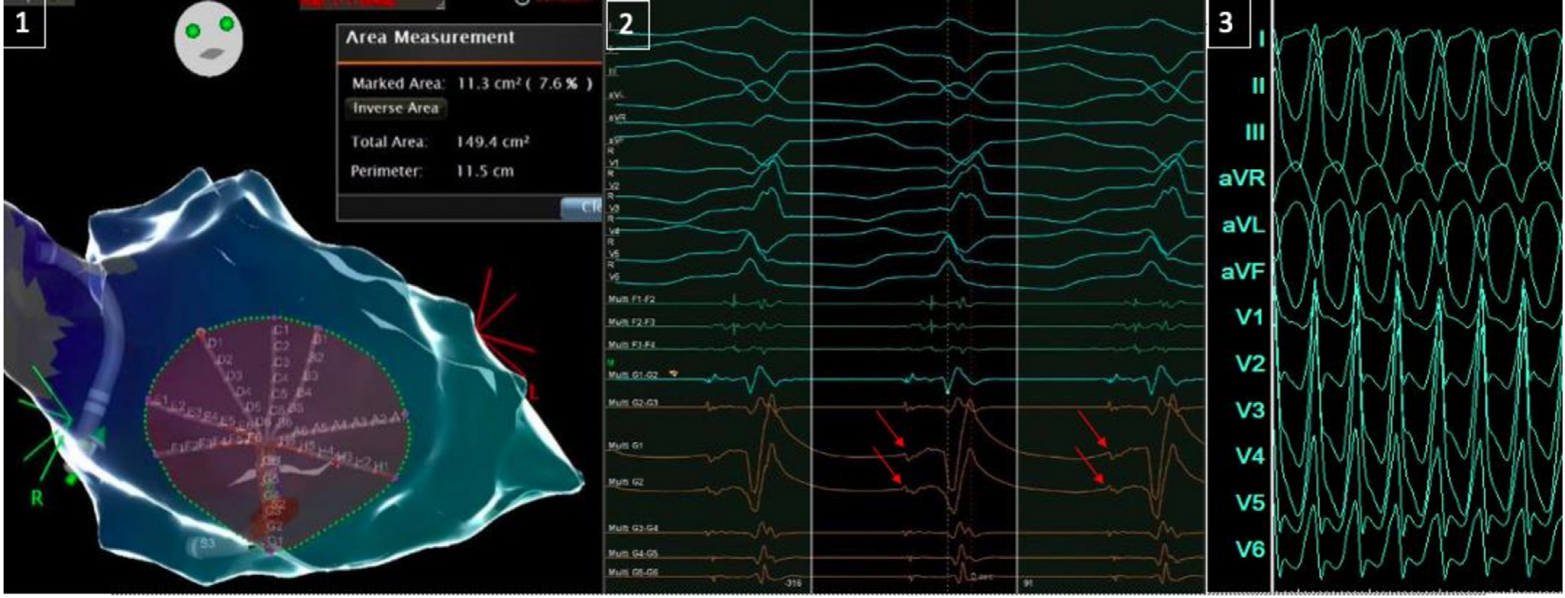
Left image (panel 1): The 8 splines of the star-shaped Octaray (Biosense Webster) stretched out equally on the surface of the left ventricular septum covering an area of >11 cm^2^ representing 7.6% of the total internal surface of the dilated left ventricle and allowing for assessing activation patterns across the splines. Middle image (panel 2): A 12-lead electrocardiogram (ECG) of ventricular tachycardia (VT-1) with inferoseptal basal exit and intracardiac electrogram examples from spline G (with clear local potentials on unipolar G1 and G2) preceding spline F, which is positioned more basally on the septum, closer to the VT exit. Right image (panel 3): A 12-lead ECG of VT-2 (25 mm/s) with inferior axis and positive concordance in the precordial leads, suggesting an exit in the anterobasal segment.

The procedure was performed under general anesthesia with transesophageal echocardiography using the CARTO® 3 (Version 7) electrophysiology mapping system (Biosense Webster). Triple right femoral venous and single right femoral arterial access was gained under ultrasound guidance and a standard quadripolar catheter placed at the right ventricular apex. A long 23 cm 8F sheath was inserted for retrograde arterial access. Trans-septal puncture was performed using a Heartspan ML1 sheath, a BRK-1 extra-sharp Brockenbrough needle, and a SafeSept wire. Unfractionated heparin was administered in boluses as per our institutional protocol to keep the activated clotting time above 300 seconds. A left ventricular substrate map during right ventricular pacing was acquired via retrograde and antegrade mapping (Figure 1A) using the 48-electrode 8-spline star-shaped Octaray mapping catheter with 2.0 cm spline length and 3-3-3-3-3 electrode configuration with the new TRUEref^TM^ technology employing an insulated unipolar electrode at the distal tip of the shaft in the center of the star-shaped splines (Figure 1C and 1D), a proprietary wavefront detection algorithm, and multichannel advanced reference annotation. Within 24 minutes an ultra-high-density map of 17,435 points was acquired (Figure 2) demonstrating a large anteroseptal scar, more extensive in the unipolar than in the bipolar map. The clinical VT (VT-1, Figure 3) was repeatedly induced by catheter manipulation over the septal area, requiring escalating vasopressor support. The variable cycle length of the VT made it impossible to collect an accurate local activation time (LAT) map with the Confidense algorithm. Using the retrograde aorta approach, the Octaray splines were optimally arrayed over a majority of the septal scar. This immediately identified the diastolic pathway electrograms, which were manually tagged. Subsequent ablation with a trans-septally inserted D-curve ThermoCool SmartTouch® ablation catheter (Biosense Webster) with 3.5 mm tip (50 W with 0.9% saline for 60 seconds with a contact force >10 g throughout) based on the electrograms (EGMs) at this site terminated the VT immediately. Consolidation lesions around this region were also delivered. During catheter manipulation a second, pulseless VT (VT-2) was induced with a morphology suggestive of a superior basal exit. Pace termination failed and external defibrillation was required on 3 occasions, thereby limiting the mapping time. Despite this, the initial high-density substrate map guided further substrate ablation extending superiorly toward the basal edge of the septal scar. The total ablation time was 54 minutes and 53 seconds. Thereafter, a VT stimulation protocol with 3 sensed extrastimuli down to 230 ms coupling interval showed noninducibility, and therefore no further ablation and substrate homogenization were pursued in the remaining areas of the scar. The underlying rhythm at the end of the procedure was sinus rhythm with third-degree atrioventricular block and no ventricular escape rhythm at 30 beats per minute. The total procedure duration was 2 hours and 32 minutes.

Beta-blockers were reintroduced the following day after extubation and successful weaning of the pharmacological hemodynamic support. No further ventricular arrhythmias were documented during inpatient monitoring. Three days postprocedure the patient was transferred back to her local hospital on beta-blockers only for further rehabilitation and reintroduction of her heart failure medication. At 3-month follow-up, she had had no further VT and her ICD was functioning normally. The postablation complete heart block had resolved.

## Discussion

Identification of a critical isthmus by demonstrating diastolic potentials^7^ and confirmation by specific entrainment criteria^8^ are still considered the gold standard for target definition in VT ablation. Hemodynamically unstable, alternating, and/or irregular VTs, as well as noninducibility, represent a significant challenge. Multiple strategies for substrate mapping have been proposed to overcome this dilemma. Traditional methods include voltage- and EGM-based approaches like late potential and local abnormal ventricular activity mapping.^9^ More recently, functional substrate mapping approaches aiming to identify zones of deceleration^10^ and decremental conduction by means of various protocols^11^ or rotational activity at baseline rhythm,^12^ frequency-based mapping techniques,^13^ and purely imaging-guided ablation^14^ have gained popularity in an effort to minimize the need for VT induction associated with a risk of hemodynamic compromise and requirement for cardioversion.

For substrate and activation mapping alike, correct detection and annotation of low-amplitude, fractionated signal close to the noise floor and overshadowed by larger far-field signals from adjacent healthier areas, with subsequent interpretation of each point in the context of its 3-dimensional neighbors as well as algorithms to account for physiological constraints, remain a challenge even for the most sophisticated mapping system. Mapping density of thousands of points make manual verification and correction in real time unfeasible, even more so if a strategy requiring multiple maps using variable wavefronts for target identification is employed.^15^

Conventional activation mapping often is a significant contributor to overall procedure time, requiring extensive, potentially proarrhythmogenic catheter manipulation and, in many cases, repeated pacing maneuvers to differentiate functionally critical areas from bystanders. Longer in-heart catheter dwell time and procedure time has been associated with higher complication rates. Prolonged pacing may cause hemodynamic instability. In turn, extensive ablation may lead to unnecessarily excessive radiofrequency delivery and risk of complications on its own.

The irrigated unidirectional (D or F curve) multispline Octaray multielectrode catheter with 48 small, tightly spaced recording electrodes comes in various configurations (2-2-2-2-2, 2-5-2-5-2, and 3-3-3-3-3 on 1.5- or 2-cm-long splines) and with an innovative, tightly referenced indifferent electrode at the tip of the shaft and 3 true-shaft electrodes enabling visualization of the shaft deflection and tip orientation. The catheter design aims to address 2 of the above-mentioned challenges—improving EGM signal quality, particularly in areas of heterogenous scar, and increasing speed of precise high-density map acquisition—all while still being integrated in the established CARTO 3 Version 7 3D mapping system. For atrial mapping procedures it was shown that the 48-electrode catheter has a 4 times higher acquisition rate, doubling the EGM points per map yet reducing mapping time by more than 50% as compared to the Pentaray in preclinical and first-in-human studies.^4^^,^^5^ Experience of its use in clinical ventricle arrhythmia ablations is scarce. Choice of Octaray spline length and electrode configuration may be guided by chamber size, substrate, and mapping strategy. The closely spaced 2-2-2-2-2 electrodes may be superior for ultra-high-resolution mapping in scar-related tachycardias, whereas larger splines have the obvious advantage of wider reach and shorter mapping times, even in significantly dilated chambers.

Our case illustrates the impressive signal quality of novel multipolar catheter designs. In particular, the unipolar signal generated between the spline poles and the isolated indifferent electrode in the shaft showed remarkably clear signal quality with little far-field artefact in the ventricle when compared with the experience of traditional unipolar references of Wilson's Central Terminal or inferior vena cava catheters. The wide reach of the Octaray’s 2-cm splines in all directions, covering large areas of the ventricular surface, facilitates near-immediate rapid diagnosis of activation patterns based on EGM interpretation alone. This facilitates mapping of unstable or even pulseless VTs, which are traditionally considered to be unmappable, by strategic positioning of the catheter over the area of interest. A further point to highlight is the incredible speed of point acquisition and density rapidly exhausting the maximum limit allowed to be displayed on the shell. Unfortunately, the longstanding issue of correct detection and LAT annotation in scar-related arrhythmias in the most critical parts of the map with high prevalence of low-voltage, fractionated EGMs remains an unresolved issue, undermining the true potential, clinical usefulness, and diagnostic value of having such high-density maps. Multiple detection algorithms in the time and frequency domain have been suggested with, so far, none achieving a fully reliable near-field annotation, particularly not in the thicker ventricular wall. The use of a dynamic window of interest, which can be repeatedly adjusted while mapping based on presence or absence of low-amplitude near-field signals, has been proposed as a pragmatic solution to facilitate correct detection and annotation in stable rhythms, yet this is not a feasible option for hemodynamically unstable arrhythmias. In summary, the sophisticated hardware and innovative design is a step in the right direction, yet there is still work to be done.

## Conclusion

The multispline multipolar Octaray mapping catheter provides excellent signal quality with particularly noticeable sharp unipolar EGM quality using the TRUEref technology and excellent catheter maneuverability. If placed strategically in areas of interest, its wide spatial reach facilitates the rapid diagnosis of VT activation pattern along the splines, allowing for mapping of even hemodynamically unstable VT.

Mapping time for ultra-high-resolution maps is short, yet a major drawback remains the failure of standard automated detection algorithms to reliably annotate near-field signals in areas of scar. This undermines the current standalone value of a high-density map for scar-related arrhythmias where manual reannotation is not feasible. Until improved and/or adjusted detection and LAT annotation approaches are integrated, judicial use of dynamic windows of interest, careful map setup, and continuous attention to LAT annotation during mapping, together with traditional EGM interpretation, remain key to guide clinical management.
